# Protein Sequences Recapitulate Genetic Code Evolution

**DOI:** 10.1016/j.csbj.2018.05.001

**Published:** 2018-05-30

**Authors:** Hervé Seligmann

**Affiliations:** Unité de Recherche sur les Maladies Infectieuses et Tropicales Emergentes, UMR MEPHI, Aix-Marseille Université, IRD, Assistance Publique-Hôpitaux de Marseille, Institut Hospitalo-Universitaire Méditerranée-Infection, 19-21 boulevard Jean Moulin, 13005 Marseille, France; The National Natural History Collections, The Hebrew University of Jerusalem, 9190401 Jerusalem, Israel

**Keywords:** Codon directional asymmetry, Genetic code structure, Gene punctuation, Secondary structure formation, Antiparallel betasheets, tRNA synthetases

## Abstract

Several hypotheses predict ranks of amino acid assignments to genetic code's codons. Analyses here show that average positions of amino acid species in proteins correspond to assignment ranks, in particular as predicted by Juke's neutral mutation hypothesis for codon assignments. In all tested protein groups, including co- and post-translationally folding proteins, ‘recent’ amino acids are on average closer to gene 5′ extremities than ‘ancient’ ones. Analyses of pairwise residue contact energies matrices suggest that early amino acids stereochemically selected late ones that stablilize residue interactions within protein cores, presumably producing 5′-late-to-3′-early amino acid protein sequence gradients. The gradient might reduce protein misfolding, also after mutations, extending principles of neutral mutations to protein folding. Presumably, in self-perpetuating and self-correcting systems like the genetic code, initial conditions produce similarities between evolution of the process (the genetic code) and ‘ontogeny’ of resulting structures (here proteins), producing apparent teleonomy between process and product.

## 1 Introduction

The structure of biological molecules includes imprints of ancient evolution at life's dawn. For example, comparisons between protein and RNA structures suggest affinities between viruses and hypothetical bacterial-like cellular ancestors (as described for protein structural families, [61], [123]; and for RNA secondary structures, [101]). The ribosome's structure testifies to even more ancient events: ribosomal protein amino acids interact preferentially with ribosomal RNA trinucleotides that correspond to that amino acid's assigned anticodon(s) according to the standard genetic code [42]. This striking fossilization of the process that determined some codon-amino acid assignments in the ribosome's structure confirms that at least some codon-amino acid assignments result from stereochemical affinities between RNA and amino acids [[118], [119], [120]].

### 1.1 Steps in the Evolution of the Genetic Code and the Translational Apparatus

Johnson and Wang [42] suggest that several processes structured the genetic code, meaning determined codon-amino acid assignments. Indeed, structurally simple amino acids tend to associate with rRNA nucleotide triplets corresponding to their genetic code codon assignments, while complex amino acids associate with their anticodons (stereochemical complexity according to Dufton [19]). This indicates a primary phase of direct codon-amino acid contact, and secondarily evolution of mRNA, anticodon and from there the proto-tRNA [97].

**Fig. 1:**
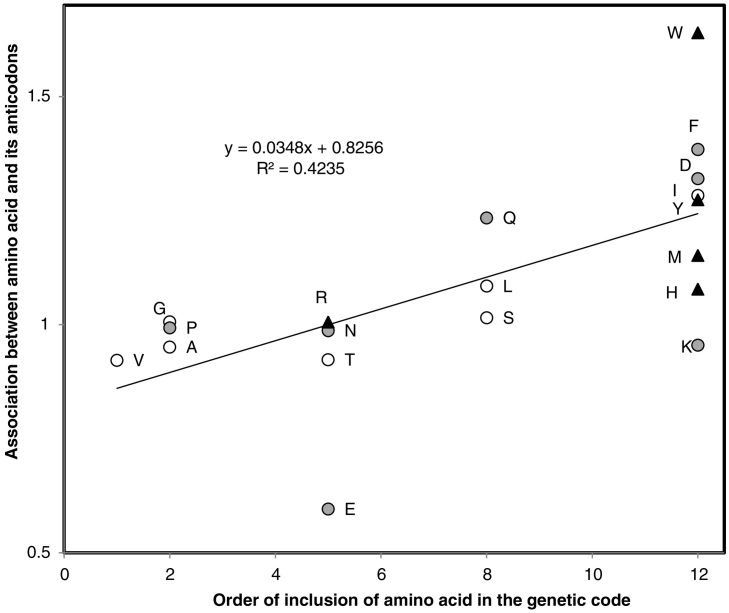
Strength of association of amino acids with ribosomal RNA triplets corresponding to their anticodons in the ribosome's structure, based on contacts between proteins and rRNAs in crystallized ribosomes [42], as a function of the order of inclusion of amino acids in the genetic code according to the tRNA Urgen hypothesis which has only 12 ranks (all ‘late’ amino acids get rank 12, [20,21,114]). Association strengths are ratios between observed numbers of amino acid contacts with anticodon triplets and expected random contacts, after data in Fig. 1 of Johnson and Wang [42]. Amino acids are classified according to three levels of structural complexity [19]: low (hollow circles), intermediate (gray circles) and high (filled triangles). The latter group would include cysteine, for which the ribosome's structure does not include contacts between residues and rRNA.

Several hypotheses predict the order of inclusion of amino acids in the genetic code. These orders tend to be consensual among hypotheses, and usually consider that structurally simple amino acids were included early, and complex one's late [36,56,113,114]. Considering 40 hypotheses about the inclusion order of amino acids in the genetic code reviewed by Trifonov [114], the strength of association between amino acids and their anticodons in rRNA (data from [42], therein figure 1) increases with their order of inclusion in the genetic code. This correlation is strongest with the inclusion order predicted by the tRNA-Urgen hypothesis ([20,21], here Fig. 1).

### 1.2 Imprints of the Genetic Code Evolution in Modern Protein Sequences

Above observations about the ribosome's structure suggest that imprints of the genetic code's evolution might remain also in protein structures. Here I test the hypothesis that the inclusion order of amino acids in the genetic code correlates with average positions of amino acids in proteins.

This working hypothesis is derived from principles of the biogenetic law or Meckel-Serres law, formulated by Haeckel as ‘ontogeny recapitulates phylogeny’ [50]. As in that evo-devo hypothesis, the history of a process might be imprinted in the structures produced by that process [44]. The reason to expect this apparent teleonomy frequently observed in biological processes is that self-organizing and self-perpetuating processes such as the genetic code are by definition self-correcting [49]. Structures resulting from early historical initial conditions are frequently conserved or recovered by resulting processes and structures. Hence historical/evolutionary processes would be conserved as imprints in modern structures because self-corrections towards the least error-prone structures conserve or recover the same initial structures/constraints. Accordingly, protein structures should also reflect the evolution of the genetic code.

### 1.3 Evolution for Coding Versatility

The genetic code evolved to include more complex amino acids, which are also more diverse in physicochemical terms than randomly selected potential amino acids [31,40,67]. Directional evolution of genetically coded amino acids towards diversification and greater complexity corresponds to the most recently integrated amino acids in the genetic code, selenocysteine and pyrrolysine [122], complex amino acids with peculiar properties (i.e. selenocysteine includes a selenium atom (doesn't occur in other natural amino acids) where cysteine has a sulfur atom (occurs only in one other natural amino acid)).

This suggests constraints towards increasing the genetic code's versatility for diverse types of specialized proteins. The evolutionary need to develop proteins with new functions would have driven inclusion of complex and physicochemically outstanding amino acids. Presumably, RNA secondary structure-based punctuation signals initiated translation before the genetic code assigned start codons [22,70]. The presumably late assignment of methionine, a structurally complex and ‘special’ amino acid, to initiation codon(s) would suggest that ‘late’ amino acids would tend to be coded close to gene 5′ extremities, and ancient amino acids closer to their 3′ extremities.

The working hypothesis expects that the genetic code evolved to include complex amino acids to stabilize protein structures, beyond increasing the diversity of potentially coded proteins. Predictions are tested versus lack of bias in average locations of amino acid species in genes/proteins.

## 2 Materials and Methods

Analyses focus on eight groups of proteins, seven from the *Escherichia coli* proteome (downloaded from GenBank entry NC_002695). Two groups consist of all tRNA synthetases of *Escherichia coli* (as used previously, [92]), subdivided in tRNA synthetase class I and class II (10 amino acid species per class, 10 proteins for class I and 13 for class II (including both subunits alpha and beta for tRNA synthetases Phe and Gly)). Class II tRNA synthetases are completed by the tRNA synthetase for pyrrolysine found in some archaea [69,109].The tRNA synthetases are chosen because these conserved proteins essential to translation occur in all organisms [66,74], including some viruses (Megavirales, [1,2,71,75]), and because within each class they are related among each other, facilitating comparative analyses [30,60,65] The two tRNA synthetase classes differ in their structures: class I are usually monomeric proteins with a Rossman fold catalytic domain. Class II tRNA synthetases are usually di- or multimeric with an anti-parallel betasheet fold flanked by alpha helices.

Other protein groups from *E. coli*'s proteome are: 67 ribosomal proteins, 36 polymerases, 119 membrane-linked proteins. Using predictions on *E. coli* protein folding modes [15], a group of 63 proteins folding cotranslationally is compared with another group of 101 proteins folding post-translationally. These were chosen from a longer protein list because predicted folding mode in these proteins does not vary with specific conditions as computationally tested by Ciryam et al. [15]. Identities and sequences of the 408 analyzed *E. coli* proteins are available in the supplementary data. The *E. coli* proteome is translated from approximately 5200 predicted genes. Excluding 1700 genes considered hypothetical according to genome annotation, the combined analyzed samples represent 9.1% of *E. coli*'s total proteome.

The last group of analyzed proteins are the thirteen, highly conserved membrane-bound proteins encoded by the human mitogenome (NC_012920, [8], [9]). Mitochondrion-encoded proteins are chosen because mitogene mutation rates have been detailed [46,47,92], enabling to test associations between errors/mutation rates and the studied variable.

**Fig. 2:**
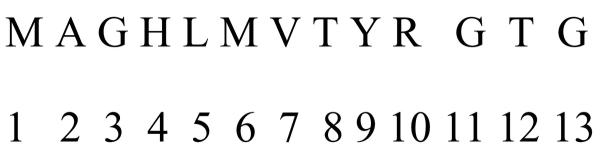
Imaginary amino acid sequence and amino acid ranked from 5′ to 3′ extremity. The mean positions are for: M, (1 + 6)/2 = 3.5; T, (8 + 12)/2 = 10: and G (3 + 11 + 13)/3 = 9. For A, H, L, V, Y and R these are 2, 4, 5, 7, 9, and 10, respectively. Further analyses of position ranks within proteins use ranks divided by the protein's total length k, here k = 13. Hence ranks divided by k range from 0.077 for the first amino acid to 1 for the last amino acid. Mean positions are also to be divided by k for comparing proteins with different sizes. For example the mean position standardized by protein size of M = 3.5/13 = 0.269.

Each residue in each protein sequence gets assigned its rank in the sequence, from 1 to k where 1 is for the first residue coded by the 5′ extremity initiation codon and k is the rank of the residue coded by the last codon before the translation termination signal. Ranks of all residues belonging to a given amino acid species are averaged, separately for each amino acid species, for each of the proteins (imaginary example in Fig. 2). This mean amino acid rank is divided by k, the total length of the protein, enabling comparisons between proteins of different lengths. For each of the 20 amino acids, a mean rank is obtained for each protein.

**Fig. 3:**
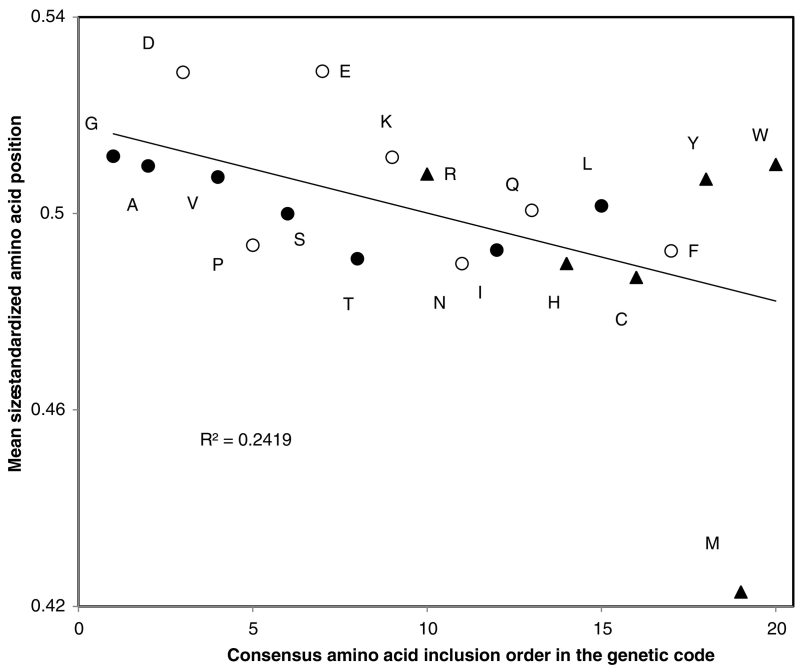
Mean position of amino acid averaged across all eight groups of selected proteins as a function of their consensus inclusion order in the genetic code [114]. Mean positions for each protein are size standardized by dividing with the protein length (y axis). The Pearson correlation *r* = −0.49, one tailed *P* = 0.014. Excluding M, *r* = −0.403, one tailed *P* = 0.044, hence the association is not due to that extreme datapoint. Amino acids are grouped according to three size/complexity levels according to Dufton [19]: low (filled circles), intermediate (hollow circles) and complex (filled triangles) amino acids. Complex/recent amino acids are on average located closer to the 5′ initiation codon than simple/early amino acids.

Ranks of inclusion of amino acids in the genetic code are those assigned by the 40 evolutionary hypotheses listed by Trifonov [114]. Statistical tests used are the sign test, using a binomial distribution assuming equal probability for each possible result, and Pearson's correlation coefficient r.

## 3 Results and Discussion

### 3.1 Biases in Mean Amino Acid Positions

For each of the 20 amino acid species A, C, D, E, F, G, H, I, K, L, M, N, P, Q, R, S, T, V, W, and Y, the mean position of residues belonging to a given amino acid species is calculated, based on the ranks of the residues in the sequence, from position 1 corresponding to the 5′ initiation codon to position k, the last codon before the termination (stop) codon. Means are divided by k, to size-standardize results (Table 1 presents results for 36 proteins, the 20 *E. coli* tRNA synthetases and the 13 human mitogenome-encoded proteins).

**Table 1:** Mean position of residues belonging to specific amino acid species in 36 proteins, divided by protein length (×100): 22 *Escherichia coli* tRNA synthetases (10 from class I and 12 from class II, indicated by their cognate amino acid), the archaean tRNA synthetases for pyrrolysine, and the 13 membrane-bound proteins encoded by the human mitogenome. Rank indicates the consensus rank of inclusion of the amino acid in the genetic code derived from the average rank of 40 inclusion orders reviewed by Trifonov [114]

	A	C	D	E	F	G	H	I	K	L	M	N	P	Q	R	S	T	V	W	Y	ro
Class I																					
Arg	62	32	51	56	43	48	39	35	45	53	49	48	47	60	54	59	45	42	60	43	−28
Cys	57	59	49	58	50	47	43	39	48	51	51	48	40	52	54	50	46	55	51	48	−2
Gln	57	46	49	51	52	51	40	45	54	50	46	46	51	44	52	51	47	60	52	42	**−45**
Glu	47	61	49	55	50	47	46	46	49	56	46	49	48	37	55	48	59	47	47	57	10
Ile	58	27	53	50	49	53	35	50	53	55	44	48	52	41	54	44	46	55	44	41	**−59**
Leu	51	35	54	50	47	51	49	49	50	51	53	45	48	51	52	44	47	59	45	42	**−47**
Met	47	53	51	54	54	49	34	49	56	46	48	48	46	48	57	55	52	45	54	56	20
Trp	59	41	47	48	44	46	52	52	54	52	44	53	47	59	46	45	51	57	44	42	**−40**
Tyr	54	18	49	61	53	51	48	41	59	49	46	48	49	43	54	54	41	56	18	43	**−50**
Val	60	57	46	52	51	47	36	53	50	58	43	51	47	46	50	47	48	48	45	39	−31

Class II																					
Ala	45	77	44	44	48	55	53	56	41	45	60	46	49	55	49	55	57	51	65	69	50
Asn	56	38	56	55	47	56	52	50	50	49	37	53	45	58	50	43	43	52	26	52	**−57**
Asp	50	78	60	48	49	58	52	60	57	45	46	47	47	48	46	38	45	52	33	52	−5
Gly α	48	36	52	56	54	51	51	53	54	53	57	52	52	38	53	40	45	42	52	60	25
Gly β	29	83	55	46	70	58	47	48	47	49	58	51	52	46	52	44	51	52	65	62	54
His	41	47	43	55	57	50	35	49	50	50	48	50	46	56	57	58	52	55	46	47	−7
Lys	50	41	46	54	56	45	47	46	53	52	43	51	44	59	54	53	49	50	47	61	12
Phe α	60	30	47	51	52	56	50	51	56	49	50	57	55	41	35	44	51	47	48	46	−26
Phe β	61	44	54	44	50	51	50	25	52	52	46	48	42	48	47	56	50	60	42	39	**−45**
Pro	47	46	58	47	51	42	42	48	48	54	59	53	43	52	54	45	46	56	33	57	−2
Ser	62	49	47	57	54	41	45	51	65	52	35	55	42	41	52	55	44	50	40	55	−12
Thr	55	35	49	51	34	54	41	50	54	54	51	45	37	53	50	51	48	57	18	35	**−62**
Pyl	40	48	62	55	65	65	37	55	43	55	55	56	45	47	46	45	37	48	62	62	19
Mito																					
AT6	47		48	63	46	45	42	51	61	55	49	41	54	65	39	48	48	61	48	51	−9
AT8		50		47	49		46	49	58	56	40	49	52	50		50	52	31	33	53	−10
CO1	54	48	58	49	49	60	54	44	37	50	50	53	45	49	50	53	49	57	49	51	**−48**
CO2	60	68	90	53	40	54	57	39		50	29	65	32	99	23	45	49	66		51	−22
CO3	52	34	64	62	39	52		46	57	54	36	27	47	62	42	60	50	57	69	50	−30
CytB	49	84	63	57	62	52	47	66	41	46	29	49	36	44	44	50	44	60	57	56	5
ND1	54		99	52	42	48	62	52	33	53	45	41	41	47	42	50	52	63	33	69	−37
ND2				76	31		71	46	66	44	35	47	55	47		84	31	12	56	59	3
ND3	37	63	45	51	53	65		47	59	47	46	53	62	45	64	52	48	56	48	52	1
ND4	44	97	47	71	53	45	53	49	63	44	51	39	50	48	49	56	51	53	50	61	32
Nd4l	36			51	48	43	46	54		57	43	50	58	38	54	48	53	35		51	17
ND5	44		61	54	59	40	72	49	35	47	46	46	46	52	55	56	57	43	60	58	29
ND6	56	23	76	63	35	49		57	37	42	31	65	59			38	66	52	71	51	−35

Rank	2	16	3	7	17	1	14	11	15	8	19	12	5	11	10	6	8	4	20	18	
Mean	51	50	55	54	50	51	48	49	51	51	46	49	47	51	50	50	49	51	47	52	
<50	14	19	15	9	17	15	21	22	15	14	25	20	24	21	14	18	22	13	20	13	
P	20	14	36	0.2	43	30	8	12	30	12	1	31	3	16	24	57	12	7	20	7	

<50 indicates the number of proteins where the mean position is <50 for that residue. The row P indicates the two tailed statistical significance multiplied by 100 according to a sign test that the number of proteins with <50 differs from random (50%). Column ro is the Pearson coefficient of correlation (multiplied by 100) between rank and the mean position of amino acids in that protein (bold indicates negative correlations with one tailed P < 0.05). >400 proteins from *E. coli*'s proteome were analyzed, Table 1 presents specific proteins of specific interests, tRNA synthetases because these are very ancient, evolutionarily interrelated protein groups, and human mitogenome-encoded proteins because their mutation rates have been extensively studied.

Considering that amino acids are either late or early according to Trifonov's consensus inclusion order in the genetic code, one tailed tests can be applied. Considering separately tRNA synthetase classes, for the most ancient tRNA synthetases (class I), results are statistically significant for H (two tailed test), A, E, I, M, N, P, R and Y (one tailed tests). For class II tRNA synthetases, P < 0.05 for C, G, and P (two tailed tests). For the 13 proteins encoded by the human mitogenome, P < 0.05 (two tailed tests) for E, M and Y.

Such analyses are also applied to five other *E. coli* protein groups. Results differ for different protein groups (Table 3). They are statistically significant at P < 0.05 according to two tailed sign tests for ribosomal proteins, polymerases, posttranslationally and cotranslationally folding proteins for five amino acids (Q, D, G, M, Q; F, M, R, V, Y; G, M, N, R, V, and G, M, N, R, V, respectively) and for eight amino acids for membrane bound *E. coli* proteins (A, C, D, E, G, L, M, Y).

### 3.2 Position Bias across Proteins and Consensual Inclusion Order

The amino acids in Table 1, Table 2 can be classified into two groups, early and recent ones (rank <11 and rank >10, respectively). According to the working hypothesis, the former should have mean positions (after size standardization according to protein length) >50, the latter <50. This is observed for the mean positions of amino acids averaged across all protein groups for 16 among 20 amino acid species. This is a statistically significant majority of cases according to a one tailed sign test (P = 0.0059). This result is confirmed by using Fisher's method for combining P values [27,28], which sums -2xlnPi where i ranges from 1 to k tests, and yields a chisquare statistic with 2xk degrees of freedom. This yields P = 5.2 × 10^−12^ for amino acids with consensus inclusion ranks <11, and P = 6.3 × 10^−35^ for those with consensus inclusion ranks >10.

**Table 2:** Numbers of proteins with mean amino acid position <50% of protein length in eight protein groups (see text) and number of analyzed proteins when that amino acid is absent from some proteins.

	Class I 10	Class II 13	Mito 13	Rib 71	Pol 36	Memb 119	Cotrans 63	Posttrans 101	All	N	P
A	2	6	6 11	**26**	15	**73**	29	44	201	423	0.238
C	6	**10**	3 8	20 33	19 32	**62** 105	28 54	41 85	**189**	337	0.012
D	6	6	3 10	**24** 70	21	**46** 117	35	42	**183**	420	0.006
E	**1**	5	2	28 69	15	**49**	31	**32**	**163**	423	0.0000037
F	4	4	9	39 67	**28**	56	34	45	219	421	0.336
G	6	**3**	6 11	**26**	20	**43**	**22**	44	**170**	423	0.0000585
H	**9**	7	4 10	41 69	18 35	52 110	32 62	47 97	210	406	0.445
I	7	5	8	37 69	17	57	31	58	220	423	0.337
K	3	4	5 11	29	13	51	27	47 100	**179**	422	0.0014
L	2	5	6	31	14	**76**	32	51	217	425	0.825
M	8	6	**11**	**55**	**25**	**82**	**46**	**90**	**323**	425	1 × 10^−40^
N	8	4	8	40	18	52	**38**	**60**	228	425	0.080
P	8	**10**	6	32 68	21	62 117	30	48	217	421	0.510
Q	6	7	7 12	**43** 69	17	59	32	54	225	422	0.110
R	**1**	5	6 10	32	**8**	57	**23**	**39** 100	**171**	421	0.00012
S	5	7	4	41 70	13 35	59	32	54 100	215	422	0.944
T	7	8	6	39 70	14	52	29	**70**	225	424	0.137
V	4	**3**	4	35 69	**24**	52	**38**	47 100	207	422	0.955
W	6	9	5 11	18 36	14 33	54 111	27 53	37 79	170	346	0.980
Y	8	4	**0**	31 63	**23** 35	**48** 117	30	43 95	187	409	0.056
R-n P	0.27 0.12	0.19 0.21	0.17 0.24	**0.62** 0.0016	0.22 0.18	0.37 0.055	0.37 0.053	0.34 0.07	**0.51** 0.011		
R-m P	**−0.55** 0.006	−0.13 0.29	−0.21 0.19	**−0.41** 0.038	−0.11 0.32	**−0.46** 0.02	−0.28 0.12	−0.31 0.10	**−0.45** 0.022		

Last column: P value (two tailed sign test). Last rows: Pearson correlation coefficients between amino acid inclusion order in the genetic code [114] and percentages of samples with mean position <50% (R-n) and with mean position divided by total length (R-m) and corresponding one tailed Ps. Bold indicates *P* < 0.05 for specific samples (sign tests).

Calculations of combined P values assume that all combined tests are independent. This author's opinion is that this is approximately correct in this case. Potential lack of independence is accounted by the correction for the mean false discovery rate α(k + 1)/(2 k), where k is the number of tests (k = 10 amino acids for inclusion ranks below 11 and those above 10) and α the critical significance level P = 0.05 [12,14,110]. The corrected critical value for combining 10 dependent statistical tests is P = 0.0275. This adjusted critical value does not qualitatively alter conclusions.

Consensus inclusion ranks of amino acids in the genetic code correlate negatively with the mean position of amino acids for each of the eight protein groups analyzed, significantly so for three among eight groups, and for all groups pooled (line R-m in Table 2). Hence mean positions of amino acids in proteins overall fits the pattern that recent amino acids tend to be positioned in the 5′ half of proteins, and early ones in their 3′ half.

### 3.3 Protein Groups and the Working Hypothesis

A statistically significant majority (eight among ten) of tRNA synthetases from class I (presumably the most ancient tRNA synthetases) have negative correlations (one tailed P = 0.0273). In five specific proteins, negative correlations have P < 0.05, none of the positive correlations has P < 0.05. For class II tRNA synthetases and proteins encoded by the human mitogenome, negative correlations are in each group non-significant majorities (7/13 and 9/13, respectively), and fewer specific correlations have P < 0.05 (class II: 3 negative and 2 positive correlations; no P < 0.05 for mitochondrion-encoded proteins).

Majorities of correlations are negative as expected also in the remaining protein groups: ribosomal proteins (53/67, P < 0.05 for 10 negative correlations), polymerases (21/36, P < 0.05 for 2 negative correlations), membrane-bound proteins (85/119, P < 0.05 for 9 negative and 3 positive correlations, respectively), co-translationally folded proteins (37/63, P < 0.05 for 7 negative and 4 positive correlations, respectively) and post-translationally folded proteins (64/101, P < 0.05 for 7 negative and 1 positive correlations, respectively). The probability to obtain majorities of negative correlations in all eight independent protein groups is P = 0.0039 according to a sign test. Majorities are significant at P < 0.05 according to sign tests for 4 among 8 protein groups, class I tRNA synthetases, ribosomal proteins, membrane-bound proteins, and post-translationally folded proteins.

Correlation strengths and directions vary widely, even within related protein groups, such as class II tRNA synthetases: r ranges from −0.53 to +0.58. Overall, recent amino acids are on average positioned in protein's first half (in relation to the gene's translational initiation region) in all protein groups chosen for this preliminary analysis. Hence the observation might be generally applicable to most proteins.

### 3.4 Mitochondrial Mutation Gradients as Potential Confounding Factors

Results in terms of associations between mean amino acid positions and their inclusion order in the genetic code are weak for the 13 mitochondrion-encoded proteins (Table 1). Their amino acid contents might be altered by mutation gradients affecting their sequences, due to time spent single stranded during light strand replication [73] and/or transcription ([105]a, [89,92,93]). Duration of single-strandedness is proportional to the combination of distances from the heavy and light strand replication origins ([105]b). This point is complicated by the fact that mitochondrial tRNAs, probably because of genome size reduction, cumulate multiple functions [32,108], including initiating replication [84,85,90,91], and transcription-initiated replication [72]. These tRNAs occasionally form OL-like structures [83,87,88,98,100,106]. Time spent single stranded is directly proportional to mutation rates, and specifically to directional mutation rates [46,47].

**Fig. 4:**
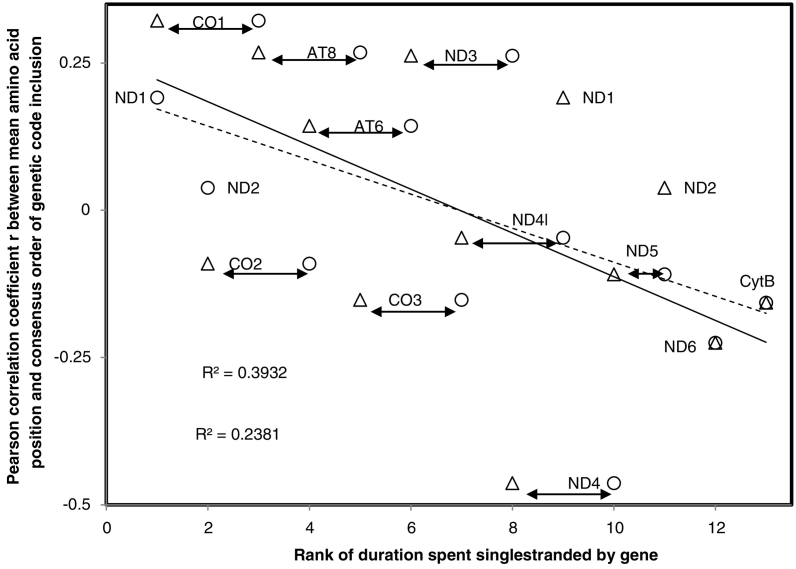
Association between mean amino acid position in the 13 human mitochondrion-encoded proteins and amino acid consensus inclusion order in the genetic code as a function of ranked times spent singlestranded by corresponding mitogenes during transcription (circles, continuous line) and during replication (triangles, discontinuous line). Mutations increase with singlestrandedness. Results indicate that amino acid mean positions reflect their genetic code inclusion order because this order increases tolerance of protein folding to mutations. This constraint affects less genes/proteins with lower mutation rates (low values for x axis). The y axis is identical for replicational and translational singlestrandedness, gene identity is indicated at mid-distance between points corresponding to replicational versus translational singlestrandedness (x axis). For ND6 and CytB, singlestrandedness is identical according to both replication and translation.

Fig. 4 plots the Pearson correlation coefficient r between inclusion order and mean amino acid position of mitochondrion-encoded human proteins as a function of their ranked time spent singlestranded during transcription (circles) and replication (triangles). This analysis yields important hints for understanding cause(s) behind the association between genetic code inclusion order and mean amino acid positions.

Firstly, singlestrandedness gradients affect strength and direction of associations between inclusion order and mean position: the expected correlation is strongest for regions of the genome with the highest mutation rates (i.e. Cytochrome B). Secondly, the transcriptional gradient is more relevant to the phenomenon under study than the replicational gradient (Transcription: r = −0.618, P = 0.012 and rs = −0.675, P = 0.0055; Replication: r = −0.485, P = 0.0465; rs = −0.554, P = 0.025, one tailed tests).

This suggests that associations between mean amino acid positions and genetic code inclusion order is more relevant to promote correct protein folding under high mutation rates, and that this translation-related phenomenon relates mainly to transcriptional errors (nucleotide misinsertions in mRNAs). A second point relates to the tendency for positive r values (y axis in Fig. 4) at low mutation rates. These (weak) positive correlations between inclusion order and amino acid positions suggest that complex residues tend to be added to elongating peptides towards the end of translation.

Analyses of the remaining seven protein groups tentatively confirm this result that the 5′-to-3′ late-to-early amino acid gradient increases folding tolerance to mutations. For each of these *E. coli* proteins, mutational rates are estimated by percentages of non-identical residues in alignments between each of the *E. coli* proteins analyzed here and their *Salmonella* homologue. The strength of the association between genetic code inclusion order and mean amino acid position correlates negatively as expected with this proxy of mutation rates in five among seven of the *E. coli* protein groups (not shown). This tendency is never statistically significant, but strengthens the status of the 3′-late-to-5′-early amino acid gradient for tolerating mutations as working hypothesis for ulterior refined analyses.

Apparently, associations between genetic code inclusion order and amino acid position prevent/decrease protein misfolding, including under high mutations. This could reflect error-preventing mechanisms in relation to protein folding.

### 3.5 The Working Hypothesis and Amino Acid Inclusion Orders in the Genetic Code

Numerous hypotheses predict the inclusion order of amino acids in the genetic code (reviewed by [114]). These follow various rationales. Ancient amino acids are presumably those found in meteorites; those spontaneously synthesized in experiments presumably reproducing conditions at earth origins; those in presumed ancient genes; amino acids with low structural complexity [19]. Other hypotheses follow more complex rationales, such as the coevolution hypothesis between amino acid and nucleotide metabolisms [116,117], and the circular code theory. The latter is based on self-correcting properties of translational frameshifts by a specific group of codons [26]. These form a circular code which enables detecting ribosomal translation frames [10,55,57]. The latter 20 circular code codons code for 10 simple amino acids among those spontaneously observed in Miller's experiment and in meteorites.

Associations between mean amino acid positions and inclusion orders according to the 40 different hypotheses listed by Trifonov [114] were calculated for each of the proteins analyzed here. For a majority of hypotheses on the inclusion order of amino acids in the genetic code, negative associations are found in >50% of proteins (for 33 among 40 hypotheses, one tailed sign test, P = 0.000001). The hypotheses with the highest numbers of negative associations between inclusion order and mean position vary according to protein groups (Table 3). Similarly, the hypothesis that yields the greatest number of statistically significant correlations (P < 0.05) between mean amino acid position in the protein and the hypothesized inclusion order also varies among protein groups.

**Table 3:** Hypotheses of amino acid inclusion order in the genetic code with strongest associations with mean amino acid positions in proteins from each protein group.

Proteins	N	Most	P < 0.05
Class I	10	Wong coevolution, Altshein-Efimov	Existing protein composition
Class II	13	Circular code	Existing protein composition
Mito	13	Hornos algebraic model	Circular code
Rbp	71	Murchison meteorite	Juke's neutral theory
Pol	28	Codon-anticodon stability	Altshein-Efimov
Membrane	120	Altshein-Efimov	N-fixing AAs first
Co	71	Hartman GC code	Existing protein composition
Post	102	Juke's neutral theory	Wong coevolution

N, number of proteins; Most, hypothesis with most correlations between inclusion order and 5′-3′ mean position of amino acids fitting the direction predicted by the working hypothesis; P < 0.05, hypothesis with most statistically significant correlations.

Overall, the Altshtein-Efimov prebiotic physicochemical code assuming stereochemical interactions between amino acids and nucleotides is the most fitting hypothesis according to Table 3, fitting previously mentioned analyses of the ribosome's structure (Fig. 1, and Johnson and Wang [42]). Other noteworthy hypotheses in Table 3 are Juke's neutral theory that assumes that the earliest amino acid were assigned to codon groups most likely to mutate into each other ([43], neutral mutation theory), Wong's metabolic coevolution hypothesis and the circular code for translational frame detection [10].

Some hypotheses in Table 3 are related. Hartman's GC code [37] and codon-anticodon interaction stability can be lumped together. Davis's hypothesis of N-fixing amino acids [17] is a metabolic hypothesis that implies a comma-free code [16], a special case among circular codes. Hence Davis's hypothesis in Table 3 strengthens metabolic and circular code hypotheses. If furthermore, one considers that some protein groups are more ancient (ribosomal proteins and tRNA synthetases), several specific factors seem to have prevalently organized the genetic code: stereochemical interactions between anticodons and amino acids, mutation neutrality, metabolic coevolution and translational frame detection/correction (circular code); high temperatures and spontaneous amino acid syntheses would have associated simple amino acids (Murchison's meteorite amino acid composition, [48]) with stable codon-anticodon (Hartman's GC hypothesis). Some protein groups might evolve for unknown reasons towards other models (mitogenome-encoded membrane proteins and the algebraic amino-acid codon symmetry model [39]).

Results suggest the possibility that several processes affected the evolution of the genetic code. All hypotheses are compatible with the idea that the genetic code evolved from early and structurally simple to late and structurally complex amino acids principle [19]. This principle is explicitly implied by the Altshtein-Efimov and to some extent Wong's hypotheses, but not directly by the complementary circular code hypothesis. Coevolution between metabolisms of nucleotides and amino acids is an attractive hypothesis as it implies the growth of metabolic networks, hence seems particularly realistic in biological terms. The complementary circular code is by far less popular despite some recent analyses that show it is not only relevant to translation, but also to frame detection during transcription [22]. The circular code theory indicates that information and error correction criteria unrelated to the amino acids themselves, but to their codons, as a group, were particularly important at early stages of the genetic code's genesis.

The natural circular code enables to retrieve the ribosomal translation frame, and apparently regulates programmed frameshifts [3,4]. Its structure associates with very deep (ancient) properties of the genetic code, such as differences between amino acid L and D enantiomers, suggesting that the circular code was part of the very first phenomena that structured the genetic code's codon-amino acid assignments [56] (all amino acids coded by genes are L enantiomers, hence the genetic code selected for these from early on). The molecular mechanisms by which the circular code regulates frame retrieval remain unknown. Conserved trinucleotide motifs belonging to the natural circular code in tRNAs [53,54] and ribosomal RNAs [23,24] suggest that these tRNAs and rRNA motifs are involved in frame retrieval and/or frame maintenance.

Juke's neutral mutation hypothesis and the circular code theory stress self-correction at two independent levels as organizing principles of the genetic code. This strengthens the working hypothesis that conserving the evolutionary inclusion order in the genetic code of amino acids in their the mean positions in proteins promotes protein folding that tolerates mutations. Preventing protein misfolding has already been suggested to have affected the genetic code's codon-amino acid assignments [[33], [34], [35]], also in relation to cotranslational protein folding [102].

### 3.6 Amino Acid Pair Contact Energy in Protein Folding and Genetic Code Inclusion Order

The emerging working hypothesis suggests that associations between mean positions of amino acids and their rank of inclusion in the genetic code reflect protein folding constraints. For that reason, analyses below explore associations between amino acid inclusion order in the genetic code and contact energies between amino acid pairs. Analyses compare results obtained for several different matrices of pairwise amino acid contact energies: the matrix P from Dosztányi et al. [18] (therein Table 2), designed to distinguish between ordered and disordered proteins; the Miyazawa-Jernigan contact energy matrix [58,59,111], adjusted for exposed (hydrophilic) and buried (within protein, hydrophobic) environments [121]; and the transformation of the Miyazawa-Jernigan matrix using Thr as a reference [13]. The Miyazawa-Jernigan matrix uses frequencies of observed pairwise residue contacts in protein structures as a proxy of contact stability. Dosztanyi et al. [18] recalculated these considering amino acid frequency biases, effectively differentiating ordered from disordered (random) proteins lacking a fixed preferred structure.

In this context, the working hypothesis suggests that the genetic code inclusion order of amino acids correlates with pairwise interaction energies between amino acids. Presumably, amino acids were included to increase protein structure stabilities.

For that purpose, Pearson correlation coefficients were calculated between the absolute value of the differences in inclusion orders of pairs of amino acids (|k1-k2|, where k is the consensus inclusion order of amino acids 1 and 2) and folding contact energies in protein structures according to these contact energy matrices, and according to the difference between the contact energies within proteins and those for the same residue pair in exposed polar environment. The working hypothesis expects that early amino acids selected late amino acids that stabilize proteins by pairwise contacts, tentatively expecting negative correlations.

Focalizing on each specific amino acid species, twenty correlations between contact energies and absolute values between differences in amino acid inclusion orders in the genetic code are calculated for each matrix: one correlation per amino acid species (one correlation per line in contact energy matrices), for its 19 contacts with other amino acid species. These correlations use 19 pairs formed by each amino acid with the remaining 19 amino acids, correlation coefficients for each of the five contact energy matrices are in Table 4.

**Table 4:** Pearson correlation coefficient r (×100) between pairwise residue interaction energies and the absolute difference between inclusion ranks of interacting residus.

AA	Dosz	Zeng et al. Intra inter delta			Bet
A	−37	36	58⁎	−25	16
C	1	17	−22	38	16
D	−44⁎	8	17	−13	6
E	−8	23	33	−13	9
F	30	48⁎	43⁎	−7	18
G	−28	44⁎	37	30	30
H	12	−7	24	−24	−24
I	−19	−26	−17	−21	−29
K	−22	−19	−31	22	−30
L	−51⁎	−42⁎	−43⁎	−5	−36
M	42⁎	45⁎	43⁎	−19	35
N	−21	−1	−17	14	1
P	−66⁎	−31	−39	−2	−36
Q	16	3	5	1	8
R	−23	12	−21	44⁎	4
S	−54⁎	39	60⁎	−8	36
T	−54⁎	55⁎	58⁎	−7	40⁎
V	−40⁎	−26	−6	−35	−20
W	46⁎	41⁎	45⁎	−38	11
Y	51⁎	62⁎	58⁎	−32	−17

Analyzed matrices of interaction energy: P matrix that distinguishes ordered from unordered proteins [18]; the refined Miyazawa-Jernigan matrices for intra- and interprotein environments (hydrophobic versus hydrophilic, [121]) and their difference (intra-inter); and the Betancourt-Thirumalai transformation of the Miyazawa-Jernigan matrix [13].

⁎ Indicates P < 0.05, two tailed tests.

#### 3.6.1 Matrix P

**Fig. 5:**
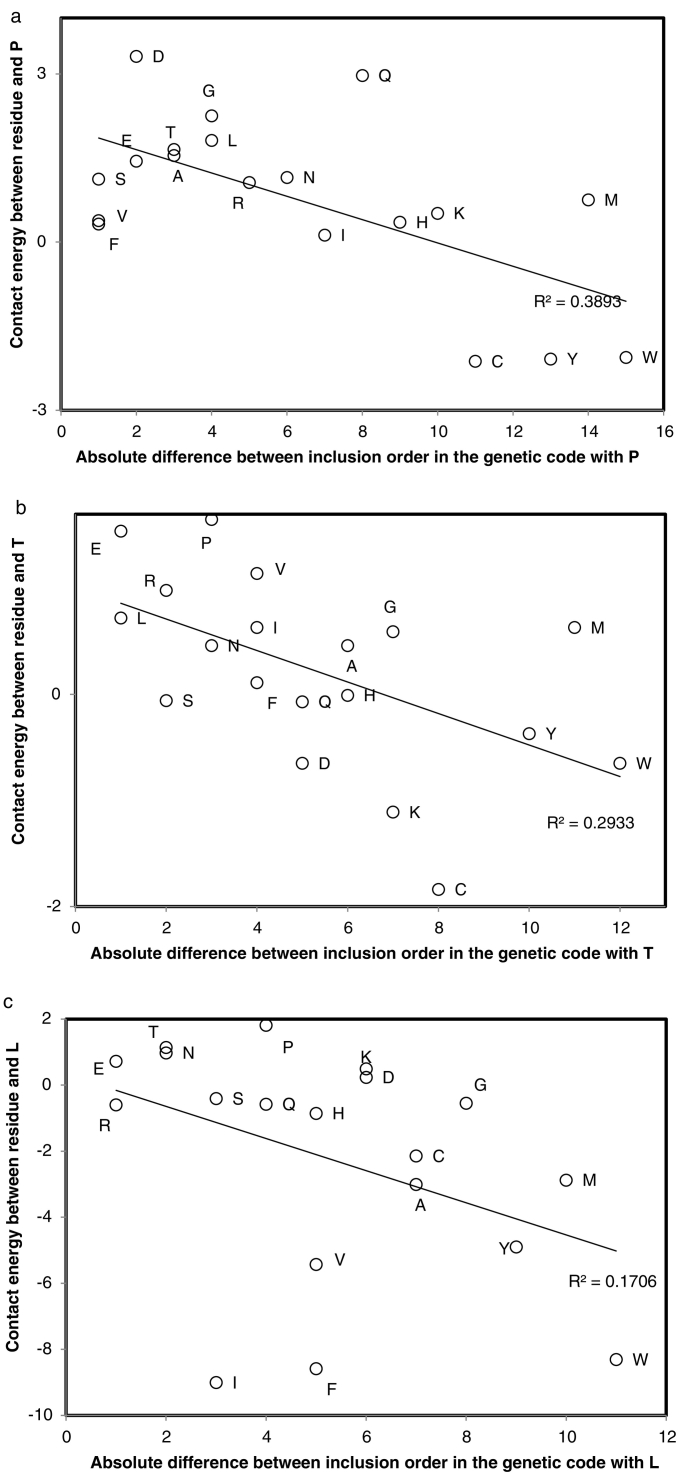
Contact energy between pairs of amino acids in folded proteins (estimated by Dosztanyi et al. 2005, therein Table 2, matrix P) as a function of absolute difference in the consensus inclusion order in the genetic code [114]. Focal amino acids are: a, proline; b, threonine; and c, leucine. Similar correlation analyses were done for each of the 20 amino acids. A statistically significant majority of correlations are negative as expected if late amino acids were selected to stabilize protein structures (16 among 20, *P* = 0.003, one tailed sign test). Three specific correlations, for amino acids T, P and W, are shown among the six that have *P* < 0.05 (Table 4).

**Fig. 6:**
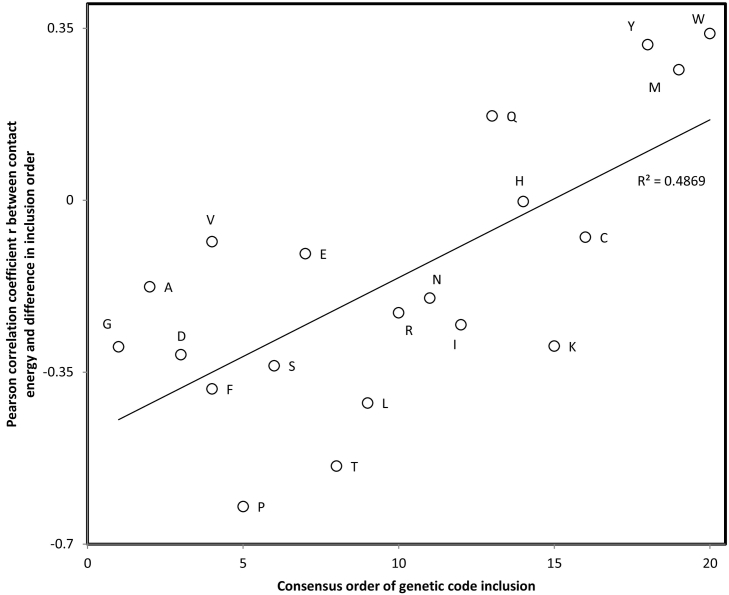
Pearson correlation coefficient r between contact energy of residue pairs and absolute difference in consensus order of inclusion in the genetic code for the pair of amino acids as a function of the consensus order of inclusion in the genetic code of the focal amino acid indicated near datapoints. For each amino acid species, an analysis such as the three presented in Fig. 3 for P, T and L are used to calculate r, which is plotted on the y axis of Fig. 3. This result shows that ‘late’ amino acids were chosen for stable contacts with early amino acids. This requirement to accommodate stereochemical moieties for several simple amino acids probably lead to the complexification of amino acid structures.

For matrix P [18], six specific negative associations have one tailed P < 0.05 (three examples in Fig. 5), for D (r = −0.44, P = 0.03), L (r = −0.51, P = 0.013), P (r = −0.66, P = 0.001), S (r = −0.54, P = 0.009), T (r = −0.54, P = 0.008), and V (r = −0.40, P = 0.045). This suggests that additions of recent amino acids stabilized proteins formed by ancient amino acids (Fig. 5). The opposite is true for late amino acids (Fig. 6). To account for the fact that these correlation tests are not independent, the correction for the mean false discovery rate α(k + 1)/(2 k) with k = 20 is applied, correcting the critical value α from P = 0.05 to P = 0.02625. Applying this adjusted criterion, correlations remain significant for L, P, S and T. Using Fisher's method to combine the 20 P values yields a combined P = 0.00009.

Results in Fig. 5, Fig. 6 are far from trivial. They mean that ‘late’ amino acids were recruited for their ability to form stable stereo- and physicochemical interactions with early amino acids. This required several moieties to accommodate different types of early amino acids (different charges, different hydrophobicities), resulting in selection for complex amino acid structures. Hence in an early phase of the genetic code, recruited amino acids were structurally simple amino acids that are frequently spontaneously synthesized as shown by Miller's experiment and amino acids found in meteorites. The later inclusions resulted from early amino acids selecting for rarer amino acids forming stable interactions with the early amino acids.

#### 3.6.2 The Betancourt-Thirumalai Contact Matrix

Similar analyses using the Betancourt-Thirumalai contact matrix [13] does not yield any clear indications. Only one among twenty correlations has P < 0.05 (for T), but in the direction opposite to the working hypothesis and the previous results for matrix P. The Miyazawa-Jernigan matrix and its Betancourt-Thirumalai transformation are based on observed pairwise contacts between amino acids within known protein structures. Matrix P is based on the bias between observed pairwise contacts as compared to random pairwise contacts. Hence the patterns observed for matrix P in relation to amino acid inclusion order relate to differences between ordered and disordered proteins, rather than to the sheer structure of ordered proteins as these could be inferred from uncorrected pairwise residue contact frequencies.

#### 3.6.3 Contact Energies within Protein Cores and between Proteins

Zeng et al. [121] recalculated the Miyazawa-Jernigan contact matrices for residue-residue interactions within the protein's hydrophobic core, and for residues exposed on the proteins surface to water's hydrophilic, polar environment. The first matrix reflects residue contacts within the protein's inner (hydrophobic) environment, which stabilize protein structures. The latter matrix reflects interaction energies that stabilize complexes between proteins. The pairwise interaction energy for exposed residues is more similar to matrix P (the correlation between these two matrices yields r = 0.64) than the matrix calculated for the protein core (r = 0.38), but both matrices for exposed residues and those within the protein's core tend to resemble each other (r = 0.73). Overall, matrix P is not too different from the Miyazawa-Jernigan-derived matrices.

Despite this similarity between matrix P and the Miyazawa-Jernigan matrices, associations between pairwise contact energies for the Miyazawa-Jernigan-derived matrices and amino acid inclusion order are in the direction opposite to that observed for matrix P. According to the Miyazawa-Jernigan matrix, contacts between amino acids with similar inclusion ranks in the genetic code tend to stabilize proteins (meaning positive correlations between contact energies and the absolute difference between amino acid inclusion orders). This is opposite to trends observed for matrix P (Table 4).

Indeed, for non-significant majorities of associations calculated for the Miyazawa-Jernigan-derived matrices, 13 among 20 correlations are positive for contact energies within protein cores, and 12 among 20 correlations for exposed residues. Among these, six and seven positive correlations have two-tailed P < 0.05 ((P values between parentheses, * after correcting the critical value α from P = 0.05 to P = 0.02625 for multiple dependent tests) within protein core: F (0.018*), G (0.03), M (0.027), T (0.008*), W (0.039) and Y (0.0025*); and for exposed residues: A (0.005*), F (0.035), M (0.033), S (0.003*), T (0.005*), W (0.025*), and Y (0.004*)). For contact energies in the protein core, the only negative correlation with P < 0.05 is for L (0.037). For exposed residues, two negative correlations have P < 0.05, L (0.034) and P (0.049) (Table 4). Hence after adjusting for dependencies between multiple tests, three and four positive correlations remain statistically significant for each exposed and protein core contact matrices, respectively. The few negative correlations are not statistically significant under these adjusted criteria. Fisher's method for combining P values yields combined P = 0.000332 (protein core) and P = 0.000026 (exposed residues).

#### 3.6.4 Linking P and Miyazawa-Jernigan-derived Matrices

Analyses of the structure of pairwise contact energy matrices in relation to the genetic code inclusion order of amino acids yield different results when considering the Miyazawa-Jernigan matrix that relates straightforward to pairwise contact frequencies versus matrix P which adjusts for amino acid frequencies, distinguishing unordered versus ordered proteins. While both types of matrices are to some extents similar, results in relation to amino acid inclusion in the genetic code tend to be opposite. For the Miyazawa-Jernigan matrix, contact stability is greatest between amino acids with similar inclusion ranks. After correction for amino acid frequencies, the opposite is observed for matrix P: contact stability tends to increase for amino acid pairs that have very different inclusion ranks.

The latter result can be obtained also for another simple transformation of the Miyazawa-Jernigan matrix, which gives us further insights into the evolution of inclusion of amino acids in the genetic code.

The two Miyazawa-Jernigan matrices derived by Zeng et al. [121] to describe interactions on exposed polar protein surfaces and in hydrophobic protein cores are used to calculate a new matrix, subtracting contact energies for the polar environment from that for the hydrophobic (protein core) environment for each of the residue pairs. For a majority of amino acids, these (signed) differences correlate negatively with the absolute value in inclusion order (for 14 among 20 amino acids, one tailed P = 0.029, Table 4). None of these specific correlations has P < 0.05, but the general tendency suggests that for contact energies in protein cores, as compared to the same, but exposed residue pair, stabilities increase with absolute differences between inclusion orders. Hence contrasting contact energies between protein surface and protein core produces, for the protein core, results reminding those obtained for matrix P, which ‘contrasts’ ordered with random proteins.

#### 3.6.5 P Versus Miyazawa-Jernigan-derived Matrices and the Genetic Code

Both patterns, that observed for Miyazawa-Jernigan matrices and that observed for matrix P, might together cause the 5′-late-to-3′-early amino acid gradients generally observed in protein sequences. Alternatively, relative dominance of constraints related to one of these matrix types could prevent or invert that gradient. Results about Miyazawa-Jernigan-derived matrices for protein cores versus protein surface suggest that potentially relevant constraints in this context are the relative importances of a protein's own stability, versus that of the interactions it forms with other proteins. Perhaps most proteins follow the 5′-late-to-3′-early amino acid gradient as a result of constraints on their own stability, while for some, perhaps relatively short proteins, the gradient is nonexistent or its direction inverted because these proteins' major constraint is to stabilize interactions among protein complexes.

Another plausible explanation is that the 5′-late-to-3′-early amino acid gradient characterizes ordered proteins, while it is lacking in disordered proteins. Indeed, results for membrane-bound proteins (very ordered proteins) suggest this approach as a valid working hypothesis. Merging membrane-bound proteins from *E. coli* with the 13 proteins from the human mitochondrion, 94 among 132 membrane-bound proteins (71.2%) qualitatively follow the 5′-late-to-3′-early amino acid gradient. This percentage is only lower than that for class I tRNA synthetases and ribosomal proteins. Ulterior tests could verify these potential refinements of the analyses presented here, using information about disordered domains in proteins [68].

In short, contacts among amino acids included in the genetic code during the same period favor protein-protein interactions. This probably reflects in a first phase, at the origins of the organic system, agglutinations of early, simple amino acids. This phenomenon would occur within water's polar environment, and would relate directly to frequencies of amino acids and hence to uncorrected pairwise contact frequencies. Today this phenomenon still occurs between residues interacting at protein surfaces, frequently between proteins. In a second phase, the short unstructured peptides required to be lengthened, and the more complex structures required stabilization. This phenomenon would have created patterns observed in analyses of matrix P: for amino acid pairs interacting within protein cores, stable interactions are between early-late/simple-complex amino acid pairs. Hence the need to stabilize complex protein structures selected late/complex amino acids in the genetic code. This complex interaction between contact energies and genetic code history presumably caused the correlation between mean amino acid positions in proteins and their inclusion order in the genetic code.

These results mirror that anticodons of complex/late amino acids interact with their amino acid in the ribosome's structure (Fig. 1), but not simple/early amino acids. Hence late amino acids had to accommodate their anticodons and neighboring amino acids. This also implies that structurally complex amino acids interacted with codons of early amino acids.

### 3.7 Molecular Evolution of Mitochondrion-encoded Proteins and Temperature

The association between mean amino acid position and the amino acid's genetic code inclusion order presumably reflects constraints for channeling protein folding towards correct folds. Temperature reflects molecular noise/unstructured movements which might render proper folding more difficult. This predicts for homologous proteins from organisms with different preferred temperatures stronger associations between amino acid inclusion order and their mean position in the organism with higher preferred temperature.

This prediction is tested for the 13 lepidosaurian mitochondrion-encoded protein genes, as listed previously [99], completed for lepidosaurian mitogenomes that became available in GenBank since 2013, and for which preferred body temperatures are available [52]. Overall, results weakly fit the prediction that high preferred body temperatures associate with stronger associations between mean amino acid positions and their order of genetic code inclusion, especially for the larger protein-coding genes. However, for some genes, the opposite seems true. More detailed analyses for specific, well known proteins by a team specialized in protein science along the lines described here will probably more adequately address this issue. Notably, coevolution between temperature and this property presumably associated with protein-folding is weaker than that observed between temperature and other molecular properties, related to transcription and translation [95,99,102].

Similarly, channeling of protein folding to avoid misfolding could also increase developmental stability. Lepidosaurian and mammalian estimates of developmental stability, estimated by fluctuating asymmetry in bilateral morphological characters (data from [77,78,80,81,103,104,107]), coevolve with several properties that presumably reflect molecular stability (mitochondrial ribosomal RNA mutational stability, [82]; mitochondrial light strand replication origin stability, [98]), or error correcting during mitochondrial translation (tRNA misloading, [11,86,[89], [90], [91],^94^]; and ribosomal frameshifts, [[84], [85], [86], [87], [88],^94^]).

Analyses indicate weak coevolution between developmental stability and associations between mean amino acid position and its inclusion order in the genetic code. These associations are stronger than those reported above for temperature, but weaker than for previously mentioned ones between developmental stability and translation-associated molecular stability/self-correcting properties. Hence the association between mean amino acid position in proteins and their genetic code inclusion order might result from properties associated to the whole organism's phenotype (preferred temperature and developmental stability). This link could explain the tendency to observe late amino acids closer to the 5′ extremity of genes and early amino acids closer to their 3′ extremity.

### 3.8 Teleonomy Between Genetic Code Structure and Protein Folding

Analyses in previous sections suggest that structurally simple amino acids that form spontaneously and were presumably the first integrated in organic life-like systems selected (in the physico- and stereochemical sense) structurally more complex amino acids that were included in the genetic code at ulterior phases. These were apparently selected for complex structures able to form stable interactions with the earlier amino acids, in particular within hydrophobic protein cores. This simple mechanism for structural stability of proteins presumably determined the inclusion order of amino acids in the genetic code.

This constraint presumably affects folding of modern proteins, so that the genetic code inclusion order of amino acids can be (on average) detected in protein sequences. This mean order of amino acids might prevent misfolding and increase tolerance of proper protein folding to mutations. Error-correcting of protein folding would be the constraint resulting in teleonomy between the genetic code's integration order of amino acids and the mean positions of amino acids in modern protein structures.

Self-organization of structures would reflect the history/evolution of the process that produced the parts of the structure, as observed for hierarchical resource partitioning in ecological communities, which tends to parallel the evolutionary relations between the species that compose the community and partition among them resources [79].

In the context of salt-adaptation of *Sorghum bicolor* [6,7], a similar property was called adaptive determinism: with no apparent sign of natural selection, most plants in a population opt for the most adapted developmental trajectories [[76], [77], [78],^96^]. The common principle for these various examples (animal embryogenesis, plant adaptation, ecological communities, and protein sequences) where teleonomy is observed would be that self-organizing processes tend towards error-correcting, self-stabilizing structures tolerating perturbations.

### 3.9 Co-versus Posttranslational Protein Folding

This observation that protein sequences frequently reflect the history of codon-amino acid assignments suggested another non-equilibrium, ‘historical’ process, cotranslational protein folding as a potential explanation for the observed pattern. Cotranslational protein folding [25,29,38,51,62,64,112,115] means that elongating, yet incomplete peptides fold during ribosomal translation, usually after specific steps in their synthesis [63]. Cotranslational folding is presumably the most ancient protein folding mechanism [41,45].

Unsurprisingly, protein folding constraints are detectable within the genetic code's structure [[33], [34], [35]]. This hypothesis predicted that sequences of cotranslationally folded proteins should follow the 5′-late-to3′-early amino acid gradient, and posttranslationally folded ones would less fit that gradient. Results comparing these two types of proteins do not fit these predictions.

## 4 Conclusions

The mean position of amino acid species tends to reflect their consensus order of inclusion in the genetic code, with early amino acids located towards the gene's 3′ extremity, and late amino acids located on average towards the gene's 5′ extremity.

Specifically, amino acid locations fit best inclusion order according to four hypotheses about the genetic code's origins: Juke's neutral mutation theory, the circular code theory [10], the metabolic coevolution theory [116] and the Altshtein-Efimov prebiotic physicochemical code based on amino acid-nucleotide interactions [5]. The two first hypotheses assume self-correcting/error-prevention principles.

These hypotheses about the genetic code's origins have very different premises: mutation neutrality, regulation of translation frame (circular code), cellular metabolism, and basic amino acid and nucleotide stereochemistry. This suggests that all four types of constraints affected amino acid inclusions in the genetic code.

The association between mean amino acid position and their genetic code inclusion order might reflect early constraints towards including amino acids that diversify the properties of proteins potentially coded by the genetic code. Here preliminary evidences suggest that this association improves chances for correct protein folding despite potential mutations, hence a potential error-correcting mechanism for protein folding.

Stability of interactions between amino acid pairs is greatest for pairs with similar ranks of inclusion in the genetic code when exposed on the protein's surface, a polar environment. This phenomenon might have promoted amino acid recruitments in the genetic code based on their capacity to agglutinate as single amino acids, or as parts of short peptides, at the earliest phases of organic systems. When peptides became longer and started folding into precise ordered structures, interactions within protein cores became more important. Indeed, when taking into account amino acid frequencies, analyses show that interactions between early and late amino acid pairs (mainly those within hydrophobic protein cores) stabilize protein structures. These observations suggest that associations between mean positions of amino acids in proteins and their genetic code inclusion order stabilize protein structures and perhaps decrease misfolding.

## Conflicts of Interest

The author declares no conflict of interest.

## Appendix A Supplementary data

Supplementary material Image 1
